# Supplementary material for: A new metriacanthosaurid theropod dinosaur from the Middle Jurassic of Yunnan Province, China
Source: PeerJ. 2025 Apr 2;13:e19218. doi: 10.7717/peerj.19218 (PMC11971988; doi:10.7717/peerj.19218)
Supplement: Supplemental Information 1 [file peerj-13-19218-s001.pdf]

Supplemental File S1

List of characters used in phylogenetic analysis. Characters are mainly sourced from matrix of Carrano et al. (2012), with modified and new characters labeled in the bracket.

Skull

1. ***Premaxilla***, inter-premaxillary suture in adults: open (0), fused (1).

2. ***Premaxilla***, height/length ratio of main body ventral to external naris: 0.5–2.0 (0), < 0.5 (1), > 2.0 (2).

3. ***Premaxilla***, ventral border of naris: subnarial process contacts nasals, excluding maxilla from narial margin (0), subnarial process separated from nasals by maxillary contribution to narial margin (1). (modified)

4. ***Premaxilla***, posterior extent of nasal process relative to posterior tip of subnarial process: even (0), posterior (1).

5. ***Premaxilla***, form of premaxilla-nasal suture: V-shaped (0), W-shaped (1).

6. ***Premaxilla***, proportions and position anterior to external nares: shorter than premaxilla ventral to nares, angle between anterior and alveolar margins > 75° (0), longer than body ventral to nares, angle < 70°, external naris overlaps some of the premaxillary body (1), external naris entirely posterior to premaxillary body (2).

7. ***Premaxilla***, diastema (‘subnarial gap’) adjacent to maxilla along dentigerous margin: absent (0), present (1).

8. ***Premaxilla***, mediolateral constriction of posterior portion: absent (0), present (1).

9. ***Premaxilla***, development of maxillary process: well-developed (0), reduced to a short triangle (1).

10. ***Premaxilla***, morphology of subnarial foramen: distinct foramen (0), expanded channel (1).

11. ***Premaxilla***, articulation with maxilla in lateral view: planar, angled from horizon smaller than or around 45° (0), planar, angled from horizon around 60° (1), planar, subvertical (2), interlocking (3). (modified)

12. ***Premaxilla***, orientation of tooth row: strongly parasagittally (anteroposteriorly) (0), mesial half teeth oriented more transversely with remnant distal still oriented parasagitally (1). (modified from Lamanna et al., 2020: 13; Brusatte et al., 2010: 10)

13. ***External naris***: long axis length: less than 50% anteroposterior diameter of the orbit (0); more than 50% anteroposterior diameter of the orbit (1). (Brusatte & Sereno 2008: 1; Holtz et al., 2004: 51)

14. ***Maxilla***, development of anterior ramus and its relationship with ascending ramus: confluent with ascending ramus or anterior ramus very short (0), offset from the base of ascending ramus, approximately as long as tall (1), offset from the base of ascending ramus, being longer than tall (2). (modified)

15. ***Maxilla***, orientation of anteriormost alveolus: vertical (0), angled anteriorly (1).

16. ***Maxilla***, shape of ascending ramus: smooth curve or straight (0), abruptly changes orientation (1).

17. ***Maxilla***, morphology of palatal process: long, ridged or fluted prong (0), long and plate-shaped (1).

18. ***Maxilla***, position of palatal process: ventral, immediately dorsal to paradental plates (0), dorsal, immediately ventral to dorsal surface of maxillary anterior ramus (1).

19. ***Maxilla***, anterior end of junction between medial wall and paradental plates: horizontal (0), inclined anteroventrally (1).

20. ***Maxilla***, horizontal ridge (prominent ‘lingual bar’) between palatal process and antorbital fenestra: absent (0), present (1).

21. ***Maxilla***, depth of paradental plates relative to anteroposterior width: low, < 1.8 (0), tall > 1.8 (1).

22. ***Maxilla***, ventral extent of paradental plates relative to lateral wall: as far ventral (0), fall short (1).

23. ***Maxilla***, arrangement of nutrient foramina on lateral surface: single row or no distinct pattern (0), two parallel rows (1).

24. ***Maxilla***, anteroventral border of antorbital fossa: graded or stepped (0), demarcated by raised ridge (1).

25. ***Maxilla***, anterior margin of antorbital fossa: rounded (0), squared (1).

26. ***Maxilla***, ventral extent of antorbital fossa: moderate (0), absent (1), dorsoventrally deep (2).

27. ***Maxilla***, position of anterior end of antorbital fossa relative to naris: posterior (0), ventral (1).

28. ***Maxilla***, development of maxillary fenestra: absent (0), fossa (1), fenestra (2). (modified)

29. ***Maxilla***, development of promaxillary fenestra: absent or fused with maxillary fenestra, no discrete fenestra (0), fenestra (1), present and extends into anterior ramus as a canal, obscured by lateral lamina (2). (modified)

30. ***Maxilla***, position of anteromedial process: ventral, immediately dorsal to interdental plates (0), dorsal, immediately ventral to dorsal surface of maxillary anterior ramus (1). (Bensen, 2010: 18)

31. ***Maxilla***, development of pneumatic fossa (excavatio pneumatica) in ascending process: absent (0), present (1).

32. ***Maxilla***, pneumaticity on medial side of posterior section of ascending ramus: absent (0), present (1).

33. ***Maxilla***, posterior end of tooth row relative to orbit: beneath (0), anterior (1).

34. ***Maxilla***, articulation with jugal: slot or groove (0), lateral shelf (1).

35. ***Maxilla***, anteroposterior length of jugal contact relative to total jugal length: less than 50% (0), more than 50% (1).

36. ***Maxilla***, ornamentation on lateral surface: absent (0), sculptured by grooves and pits (1). (modified from Lamanna et al., 2020: 24; Foster, 1999)

37. ***Maxilla***, curvature of jugal ramus: straight (0), ventrally curved (1). (modified from Eddy & Clarke 2011: 15; Holtz et al., 2004: 32)

38. ***Nasal***, inter-nasal contact in adults: separate (0), partly or fully fused (1).

39. ***Nasal***, posterior narial margin: absent or weak fossa (0), large fossa (1), laterally splayed hood (2).

40. ***Nasal***, participation in antorbital fossa: absent or at edge (0), present (1).

41. ***Nasal***, antorbital fossa in lateral view: visible (0), occluded by ventrolaterally overhanging lamina (1).

42. ***Nasal***, pneumatic foramina: absent (0), present (1).

43. ***Nasal***, development of dorsolateral surfaces: none, nasals low and dorsally convex (0), pronounced dorsolateral rims, sometimes with lateral crests (1), tall, parasagittal crests (2). (modified)

44. ***Nasal***, sculpturing: absent, surface smooth (0), low rugosity (1), deeply rugose, bears large excresences (2). (modified)

45. ***Lacrimal***, anterior process: dorsoventrally deep (0), dorsoventrally narrow, includes antorbital fossa and rim (1), dorsoventrally narrow, antorbital fossa only (2).

46. ***Lacrimal***, morphology of lateral lamina of ventral process of lacrimal: anteriormost point situated around mid-height of ventral process (0), anteriormost point situated dorsal to mid-height of ventral process and a distinct rugose patch is present on the lateral surface (1), no evident anteriormost point (2), lateral lamina forms a deep convex occupying more than half dorsoventral depth of ventral process (3). (modified)

47. ***Lacrimal***, dorsal and ventral portions of antorbital fossa: separated by anterior projection of lateral lamina (0), continuous, lateral lamina does not project far anteriorly (1).

48. ***Lacrimal***, lacrimal fenestra morphology: absent (0), present as small foramen (1), present as large oval opening with associated dorsal rugosity, swelling or ‘horn’ (2).

49. ***Lacrimal***, openings in lacrimal recess: single (0), multiple (1).

50. ***Lacrimal***, horn morphology: small rugosity (0), low, broad, rugose bar (1), triangular horn (2), dorsal projection contributed to the midline crest (3). (modified)

51. ***Lacrimal***, suborbital process: absent (0), present (1).

52. ***Lacrimal***, angle between anterior and ventral rami: ~90° (0), < 75° (1).

53. ***Lacrimal***, length of anterior process relative to ventral process: subequal (0), ~75% (1).

54. ***Jugal***, position of anterior end: posterior to internal antorbital fenestra, but reaching its posterior rim (0), excluded from internal antorbital fenestra (1), expressed at rim of internal antorbital fenestra, with distinct anterior process extending beneath it (2).

55. ***Jugal***, pneumatisation: absent (0), internally hollowed and transversely inflated by foramen in posterior rim of antorbital fossa (1).

56. ***Jugal***, antorbital fossa: absent (0), present (1).

57. ***Jugal***, morphology of lacrimal articulation: abuts, no flange (0), overlapping, flange present (1).

58. ***Jugal***, orientation of posterior margin of orbit: slightly angled posterodorsally (0), strongly angled posterodorsally, forming a gentle curve with anterior process (1), vertical (2). (modified)

59. ***Jugal***, depth of body beneath anteroventral corner of lateral temporal fenestra relative to depth of body beneath the orbit: shallower (0), subequal (1), deeper (2). (modified)

60. ***Jugal***, length of quadratojugal ramus: subequal (0), dorsal process shorter than ventral process (1). (modified from Lamanna et al., 2020: 58; Harris, 1998)

61. ***Jugal***, dorsoventral depth of postorbital ramus relative to body beneath the orbit: subequal or lower (0), prominently deeper (1). (modified from Lamanna et al., 2020: 59; Novas et al., 2013)

62. ***Jugal***, lateral view, small accessory prong between dorsal and ventral prong of quadratojugal process: absent (0), present (1). (Eddy & Clarke 2011: 34)

63. ***Postorbital***, articulation with jugal: planar (0), grooved, ventral process with U-shaped cross-section (1).

64. ***Postorbital***, suborbital flange: absent (0), present as small eminence (1), present as large flange (2).

65. ***Postorbital***, ventral extent relative to ventral margin of orbit: substantially above (0), approximately same level (1).

66. ***Postorbital***, participation in supratemporal fossa: fossa extends onto dorsal surfaces of anterior and posterior processes (0), anterior process only (1), posterior process only (2).

67. ***Supraorbital shelf*** formed mostly by ‘palpebral’: absent (0), present (1).

68. ***Orbit***, contour: subcircular (0), dorsoventrally elongate (1). (modified from Lamanna et al., 2020: 16; Smith et al., 2007)

69. ***Postorbital***, anterior prominence: absent or small (0), large (1), contacts lacrimal (2).

70. ***Postorbital***, articulation with squamosal: tongue-in-groove (0), helical (1).

71. ***Laterosphenoid***, articulations: frontal and postorbital (0), postorbital only (1).

72. ***Laterosphenoid***, location of opening for cranial nerve V: anterior to nuchal crest (0), posterior to nuchal crest (1). (modified from Lamanna et al., 2020: 73; Coria & Currie, 2002)

73. ***Prefrontal***, condition in adults: separate, moderate (0), separate, reduced (1), partly or completely coossified with other bone (2). (modified)

74. ***Prefrontal***, articulation with frontal: planar (0), peg-and socket (1).

75. ***Frontal***, exposure along orbital rim: broad (0), narrow or absent (1).

76. ***Frontal***, dorsal view, relative length of associated frontals: longer than wide (0), as wide as (or wider than) long (1). (Eddy & Clarke, 2011: 51; Smith et al., 2007: 63; Allain, 2002: 21)

77. ***Frontal***: separated from its counterpart (0), fused with its counterpart (1). (modified from Lamanna et al., 2020: 42; Holtz, 1998)

78. ***Frontal and parietal***: separate (0), fused (1). (modified from Lamanna et al., 2020: 43; Forster, 1999)

79. ***Parietal***, articulation with supraoccipital: abuts (0), overlaps (1).

80. ***Parietal***, development of median skull table: flat and broad (0), narrow with sagittal crest (1), very broad,

widely separating upper temporal fenestrae (2).

81. ***Parietal***, size and elevation of nuchal wedge and alae: moderate (0), tall and expanded (1).

82. ***Supratemporal fossa***, anteromedial corner: open dorsally (0), partially roofed over by a small shelf of the frontal-parietal (1).

83. ***Lateral temporal fenestra***, size relative to orbit: evidently smaller (0), subequal all slightly larger (1). (new)

84. ***Squamosal***, morphology of ventral process: tapering (0), bar-like with constant anteroposteriorly width (1), anteroposteriorly expanded at distal end (2). (modified)

85. ***Squamosal***, lateral view, distal end of posterior process: tapering (0), rounded (1), squared and expanded (2). (modified from Eddy & Clarke 2011: 45)

86. ***Squamosal***, lamina between medial process and anterior process: emarginated (0), continuous (1). (wording modified)

87. ***Squamosal***, flange covering quadrate head laterally: absent (0), present (1)

88. ***Squamosal***, articulation with quadratojugal: at tip (0), absent (1), broad, through ventral margin of ventral process (2), broad, through posterior margin of ventral process (3). (modified)

89. ***Squamosal***, length of ventral process: longer than posterior process (0), subequal to posterior process (1). (modified from Lamanna et al., 2020: 64; Brusatte & Sereno, 2008)

90. ***Quadratojugal***, anteriormost point of ventral process relative to lower temporal fenestra: ventral (0), anterior (1).

91. ***Quadrate***, pneumatisation: absent (0), present (1).

92. ***Quadrate***, height of dorsal ramus relative to orbit height: less (0), greater (1).

93. ***Quadrate***, axis in posterior view: vertical (0), oblique (1).

94. ***Quadrate***, height of pterygoid flange relative to complete bone: 2/3 (0) subequal (1).

95. ***Quadrate,*** quadrate foramen: absent (0), present, located between quadrate and quadratojugal (1), present, surrounded by quadrate (2). (modified from Lamanna et al., 2020: 66; Harris, 1998; Brusatte et al., 2010:108)

96. ***Quadrate***, location of mandible joint when skull is horizontally placed: approximately straight below quadrate head (0), significantly anterior to quadrate head (1), significantly posterior to quadrate head (2). (modified from Rauhut, 2003: 51)

97. ***Quadrate***, head shape in dorsal view: oval (0), subrectangular (1).

98. ***Quadrate***, medial foramina adjacent to condyles: absent (0), present (1).

99. ***Paroccipital process***, position of ventral rim of base relative to occipital condyle: above (0), approximately within its dorsal and ventral border (1), below (2). (modified)

100. ***Paroccipital process***, position of ventral edge of distal end relative to occipital condyle: at or above dorsal border of condyle, process approximately horizontal or dorsolaterally inclined (0), at or below mid-height of condyle, process ventrolaterally oriented (1).

101. ***Supraoccipital***, anteroposterior depth of median ridge relative to occipital condyle length: less (0), greater (1).

102. ***Supraoccipital***, width of knob relative to foramen magnum diameter: equal (0), 1.5x (1).

103. ***Supraoccipital***, participation in foramen magnum: absent, exoccipitals contact dorsally (0), narrow, separating exoccipitals on dorsal edge of foramen (1), wide, supraoccipital extends ventrolaterally around foramen magnum (2).

104. ***Basioccipital***, ventrolateral pair of pneumatic cavities invading neck of occipital condyle and joining medially: absent (0), present (1).

105. ***Basioccipital***, sharp dorsoventrally oriented lamina situated immediately ventral to occipital condyle: absent (0), present (1).

106. ***Basioccipital***, fossa ventral to occipital condyle in basioccipital apron: narrow and groove-like, one-half or less the width of the occipital condyle (0), broad depression approximately two-thirds the width of

occipital condyle (1).

107. ***Basioccipital***, notch along contact with exoccipital opisthotic: absent (0), present (1).

108. ***Basioccipital***, width of basal tubera relative to occipital condyle width: ≥ (0), < (1).

109. ***Basisphenoid***, location of basipterygoid processes relative to basal tubera: anterior or slightly anteroventral, basisphenoid recess opens ventrally (0), ventral, basisphenoid recess narrow and opens posteroventrally (1), anteroventrally, basisphenoid recess opens posteroventrally (2).

110. ***Basisphenoid***, depth of basisphenoid recess: shallow (0), very deep (1).

111. ***Basisphenoid***, shape of opening for basisphenoid recess: ovoid (0), teardrop-shaped (1).

112. ***Basisphenoid***, depth of indentation between basal tubera and basipterygoid processes: deep notch (0), shallow embayment (1).

113. ***Basisphenoid***, proportions of basipterygoid processes: elongate (0), broad (1).

114. ***Braincase***, number of foramina (representing cranial nerves XII, XI and X) exiting ventrolateral to occipital condyle: two (0); three (1).

115. ***Braincase***, ventral extension of subcondylar recess: pronounced (0); shallow/absent (1); narrow incisure (2).

116. 1***Braincase***, shape of ventral margin of paroccipital process and stapedial groove/foramen ovale: open curve (0); acute/closed curve (1).

117. ***Braincase***, anteroposterior angle of occiput in lateral view: vertical (0), sloping anterodorsally–posteroventrally (1).

118. ***Braincase***, morphology of trigeminal foramen: single (0), partly split (1), fully split (2).

119. ***Braincase***, median ridge separating exits of left and right sixth cranial nerves: present (0), absent (1).

120. ***Braincase***, number of tympanic recesses: two (0), three (1).

121. ***Braincase***, internal carotid pneumatization: absent (0), fossa (1), opening (2).

122. ***Braincase***, ossification of interorbital region: weak or absent (0), extensive, ossified sphenethmoid and interorbital septum (1).

123. ***Braincase***, angle between occipital condyle and basal tubera: perpendicular, at or near 90º (0), acute (1). (modified from Eddy & Clarke 2011: 64; Lamanna et al 2020: 68; Smith et al., 2007: 99; Coria & Currie, 2002: 7; Foster, 1999)

124. ***Palatine***, shape: triradiate (0), tetraradiate, well-developed jugal process (1).

125. ***Palatine***, anteroposterior extent of maxillary flange: short (0), extended (1).

126. ***Palatine***, morphology of jugal process: tapered process (0), expanded process (1).

127. ***Palatine***, orientation of maxillary contact: lateral (0), ventral (1).

128. ***Palatine***, pneumatic recess: absent (0), present (1).

129. ***Pterygoid***, pocket on ectopterygoid flange: absent (0), present (1).

130. ***Ectopterygoid***, dorsoventral depth: narrow (0), deep (1).

131. ***Ectopterygoid***, ventral fossa: absent (0), present (1).

132. ***Ectopterygoid***, lateral depth of ectopterygoid fossa: shallow (0), deep (1).

133. ***Mandible***, anteroposterior length of external mandibular fenestra: less than 15% total mandibular length (0), more than 15% total mandibular length (1). (modified from Lamanna et al., 2020: 85; Harris, 1998)

134. ***Mandible***, dorsoventral depth of external mandibular fenestra: Large, surangular above the fenestra accounts for less than half of the height of the mandible (0), reduced, surangular accounts for more than half the height of the mandible (1). (modified from Schade et al. 2023 :151;)

135. ***Mandible***, position of anterior end of external mandibular fenestra relative to last dentary tooth:

posterior (0), ventral (1).

136. ***Dentary***, shape of anterior end in lateral view: blunt and unexpanded (0), dorsoventrally expanded, rounded, and slightly upturned (1), 'squared off' in lateral view via anteroventral process (2).

137. ***Dentary***, size of mesialmost alveoli: subequal (0), third alveolus circular and enlarged (1).

138. ***Dentary***, shape in dorsal view: straight (0), curves anteromedially (1).

139. ***Dentary***, paradental groove: narrow along entire length (0), wide anteriorly defining a distinct gap between medial dentary wall and paradental plates (1).

140. ***Dentary***, longitudinal groove housing dorsally situated row of neurovascular foramina on lateral surface: absent or weak (0), present and well-defined (1).

141. ***Dentary***, number of Meckelian foramina: one (0), two (1).

142. ***Dentary***, morphology of posterior end: notched by external mandibular fenestra (0), straight or slightly concave (1).

143. ***Dentary***, morphology of surangular articulation just above external mandibular fenestra: small notch (0), large socket (1).

144. ***Splenial***, contour of posterior edge: straight (0), curved (1), notched (2).

145. ***Splenial***, size of splenial ('mylohyoid') foramen: small (0), large (1).

146. ***Splenial***, foramen in ventral part: completely enclosed by bone (0), open anteroventrally (1).

147. ***Surangular***, horizontal ridge on lateral surface below mandibular joint: weak or absent (0), strong (1).

148. ***Surangular***, number of posterior surangular foramina: one (0), two (1).

149. ***Prearticular***, mylohyoid foramen: absent or fails to perforate anteroventral margin of prearticular (0); present, foramen height less than half dorsoventral height of prearticular above foramen midline (1); present, foramen height greater than half dorsoventral height of prearticular above foramen midline (2) (Eddy & Clark 2011: 99)

150. ***Mandibular glenoid***, morphology of medial edge: flat or rounded (0), projecting (1).

151. ***Mandibular glenoid***, development of anterior wall: weak (0), tall (1).

152. ***Retroarticular process***, length: long (0), blunt (1).

153. ***Retroarticular process***, mediolateral width relative to posterior width of dentary: $\leq$ (0), $>$ (1).

154. ***Retroarticular process***, orientation of attachment surface: posterodorsal (0), posterior (1).

155. ***Paradental plates***, continuity and replacement groove: separated, groove present (0), forming a continuous medial lamina ('fused'), groove absent (1).

156. ***Paradental plates***, visibility in medial view: widely exposed, subpentagonal and moderate–tall (0), obscured by medial wall of dentary, triangular apices only may be visible (1).

157. ***Paradental plates***, surface texture: smooth (0), vertically striated or ridged (1).

158. ***Teeth***, maxillary and dentary, curvature of crown: mesial margin strongly curved and distal margin slightly curved or straight (0), mesial and distal margins strongly curved, with apex positioned well distally from distal margin (1), mesial and distal margins nearly straight (2). (modified from Lamanna et al., 2020: 8; Holtz et al., 2004)

159. ***Teeth***, crown striations: absent (0), present (1).

160. ***Teeth***, enamel wrinkles: absent (0), present, extending as bands across labial and lingual tooth surfaces (1), pronounced marginal enamel wrinkles (2).

161. ***Teeth***, mid-crown cross-section: elliptical (0), circular (1).

162. ***Teeth***, root shape: broad (0), tapered (1).

163. ***Teeth***, maxillary and dentary, serrations: present (0), absent (1).

164. ***Teeth***, maxillary and dentary, extent of anterior carina: to base of crown (0), at mid-height of crown or more

dorsally (1).

165. ***Teeth***, anteriorly procumbent anteriormost premaxillary and dentary teeth: absent (0), present (1). (new)

166. ***Premaxillary Teeth***, arrangement of carinae: nearly symmetrical, on opposite sides (0), more asymmetrical, both on lingual side (1).

167. ***Premaxillary Teeth***, serrations: present (0), absent (1).

168. ***Premaxillary Teeth***, number: four (0), three (1), five (2), six/seven (3).

169. ***Premaxillary Teeth***, spacing: even (0), paired and spaced (1).

170. ***Premaxillary Teeth***, size of tooth 1 relative to others: subequal (0), smaller (1).

171. ***Maxillary Teeth***, number: > 17 (0), 11–17 (1), < 11 (2).

172. ***Maxillary Teeth***, mid-tooth spacing: adjacent (0), with diastemata (1).

173. ***Dentary Teeth***, size and number relative to maxillary teeth: approximately equal (0), smaller and approximately 1.5 times as numerous (1).

Axial skeleton

174. ***Presacral vertebrae***, anterior articular surface of anterior centra: flat or slightly convex (0), strongly convex to be ball-like (1). (wording modified)

175. ***Presacral vertebrae***, pleurocoel posterior to parapophysis (anterior pleurocoel) in anterior elements: absent (0), present (1)

176. ***Presacral vertebrae***, posterior pleurocoel in anterior elements: absent (0), present (1).

177. ***Presacral vertebrae***, extent of anterior pleurocoel: to D4 (0), to sacrum (1).

178. ***Presacral vertebrae***, internal structure of pneumatic centra: absent, 'pleurocoels' if present, form fossae, not foramina (0), camerate (1), camellate (2).

179. **Atlas**, length of epipophyses: moderate (0), elongate (1).

180. ***Axis***, spinous process shape in anterior view: dorsal end expanded transversely (0), tapers mediolaterally (1).

181. ***Axis***, orientation of intercentrum ventral surface: horizontal or slightly anteroventral (0), tilted anterodorsally (1).

182. ***Axis***, length of epipophyses: moderate (0), long (1), short (2).

183. ***Axis***, morphology of spinopostzygapophyseal lamina: broad, well-developed (0), invaginated (1).

184. ***Axis***, development of parapophyses: moderate/large (0), reduced/absent (1).

185. ***Axis***, development of diapophyses: moderate (0), reduced or absent (1).

186. ***Axis***, pleurocoels: absent (0), present (1).

187. ***Axis***, anterior end of neural spine: evidently ascended from prezygapophysis (0), slightly dorsal to prezygapophysis (1) (new)

188. ***Cervical vertebrae***, development of pleurocoel: absent (0), a single pleurocoel (1), an anterior pleurocoel positioned posterior to parapophysis and a posterior pleurocoel positioned near the posterior margin of centrum (2), two pleurocoels positioned posterior to parapophysis and separated by a thin lamina (3). (modified from Lamanna et al., 2020: 98; Bensen et al., 2010; Gauthier, 1986)

189. ***Cervical vertebrae***, middle, shape of anterior pleurocoel: round (0), anteroposteriorly elongate (1).

190. ***Cervical vertebrae***, anterior, ventral keel: present (0), absent or weak ridge (1).

191. ***Cervical vertebrae***, anterior, demarcation of dorsal surface of neural arch from diapophyseal surface: gently sloping (0), ridge (prominent prezygapophyseal-epipophyseal lamina) (1).

192. ***Cervical vertebrae***, position of parapophysis on centrum: anterior (0), middle (1).

193. ***Cervical vertebrae***, articular surface of prezygapophyses: planar (0), flexed (1).

194. ***Cervical vertebrae***, perimeter of anterior articular

surface: not rimmed by a flattened peripheral band (0), flat, forming a distinct rim (1).

195. ***Cervical vertebrae***, anterior, transverse distance between prezygapophyses relative to width of neural canal: < (0), >, prezygapophyses situated lateral to neural canal (1).

196. ***Cervical vertebrae***, anterior, morphology of epipophyses: low, blunt (0), long, thin (1), long, robust (2).

197. ***Cervical vertebrae***, anteroposterior length of neural spines: nearly as long as centrum (0), ≤ 75% centrum length (1).

198. ***Cervical vertebrae***, longest post-axial elements: first five (0), last five (1).

199. ***Cervical vertebrae***, middle, length/height ratio of centra: less than 3 (0), more than 3 (1).

200. ***Cervical vertebrae***, posterior, neural spine morphology: low, evidently longer than wide (0), rod-like, with subequal transverse width and anteroposterior length (1), rod-like with bifurcated distal end (2), sheet-like and subrectangular (3). (new)

201. ***Dorsal vertebrae***, pneumaticity/webbing at base of neural spines: absent (0), present (1).

202. ***Dorsal vertebrae***, accessory centrodiapophyseal lamina: absent (0), present (1).

203. ***Dorsal vertebrae***, size of infraprezygapophyseal fossa: small (0), expanded (1).

204. ***Dorsal vertebrae***, anterior, ventral keel: absent or developed as a weak ridge (0), pronounced, around 1/3 the height of centrum and inset from lateral surfaces (1).

205. ***Dorsal vertebrae***, anterior, size of pneumatic foramen in centrum: small (0); enlarged (1).

206. ***Dorsal vertebrae***, elevation of parapophyses: slightly elevated from centrum (0), project far laterally, more than half the diapophyseal length (1).

207. ***Dorsal vertebrae***, morphology of hyposphene in posterior view: triangular (0), transversely narrow rectangular (1). (wording modified)

208. ***Dorsal vertebrae***, position of parapophyses in posteriormost elements: on the same level as transverse process (0); distinctly below transverse process (1).

209. ***Dorsal vertebrae***, distinct step-like ridge lateral to hyposphene, running posterodorsally from dorsal border of neural canal to posterior edge of postzygapophyses: absent (0); present (1); ridge present and is developed into a prominent lamina that bisects the infrapostzygapophyseal fossa in posterior dorsal vertebrae (2).

210. ***Dorsal vertebrae***, middle and posterior, postzygapophyses with tab-like lateral extensions of articular facets: absent (0); present (1).

211. ***Dorsal vertebrae***, morphology of neural spines: transversely compressed sheets (0), transversely broad anteriorly and posteriorly, central regions of lateral surface embayed by deep vertical troughs (1).

212. ***Dorsal vertebrae***, posterior, inclination of neural spines: vertical or posterior (0), anterior (1).

213. ***Dorsal vertebrae***, height of neural spines relative to centrum height: low, ≤ 1.3x (0), moderate, 1.4-1.8x (1); tall, ⩾ 2.0x (2).

214. ***Dorsal vertebrae***, posterior, centrum constriction: weak (0), strong (1).

215. ***Dorsal vertebrae***, centrum length relative to height: more than 2 (0), less than 2 (1).

216. ***Sacral vertebrae***, centrum pneumaticity: absent (0), pleurocoelous fossae (1); pneumatic foramina (2).

217. ***Sacral vertebrae***, number: 2 [primordial sacrals only] (0), 5 [1 dorsosacral, 2 caudosacrals] (1), 6 [2 dorsosacrals, 2 caudosacrals] (2).

218. ***Sacral vertebrae***, transverse dimensions of middle centra relative to other sacrals: equivalent (0), constricted (1).

219. ***Sacral vertebrae***, orientation of ventral margin of middle centra: approximately horizontal (0), strongly arched (1).

220. ***Sacral vertebrae***, dorsal edge of neural spines: as thin as remainder of spine (0), transversely thickened (1).

221. ***Sacral vertebrae***, pneumaticity of neural arches: weak

or absent (0), paired fossa ventral to diapophyses (1).

222. ***Caudal vertebrae***, anterior, morphology of ventral surface: flat (0), groove (1), ridge (2).

223. ***Caudal vertebrae***, L-shaped neural spines: absent (0), present (1).

224. ***Caudal vertebrae***, pleurocoels (large pneumatic foramina in centrum): absent (0), present (1).

225. ***Caudal vertebrae***, anterior, centrodiapophyseal laminae on neural arch: weak or lacking (0), as prominent as in dorsal vertebrae, defining deep infradiapophyseal fossa that penetrates neural arch (pneumatic) (1).

226. ***Caudal vertebrae***, anterior, proportions of neural arch base relative to centrum proportions: < (0), ≥ (1).

227. ***Caudal vertebrae***, middle, morphology of neural spines: rod-like and posteriorly inclined (0), subrectangular and sheet-like (1), rod-like and vertical (2).

228. ***Cervical ribs***, articulation with cervical vertebrae in adults: separate (0), fused (1).

229. ***Cervical ribs***, length of anterior process: short (0), long (1).

230. ***Gastralia***, posteriormost gastral segments: separate (0), united into single, boomerang-shaped elements (1).

231. ***Sacral ribs***, articulations in adults: separate (0), fused together (1).

232. ***Sacral ribs***, position of posterior attachment to ilium: ventral (0), posterodorsal (1).

233. ***Sacral ribs***, depth relative to ilium height: < 85% (0), ≥ 90% (1).

234. ***Chevrons***, morphology in middle caudal vertebrae: rod-like or only slightly expanded ventrally (0), L-shaped (1).

235. ***Chevrons***, proximal articular surface: divided into anterior and posterior facets by distinct transverse ridge (0), no ridge, but low lateral mounds may be present, one on each side (1).

236. ***Chevrons***, curvature: straight or gently curved (0), strongly curved (1).

237. ***Chevrons***, anterior process: absent (0); present (1).

238. ***Chevrons***, morphology of distal end in anterior and middle elements: expanded anteroposteriorly (0), unexpanded, tapers ventrally (1).

Appendicular skeleton

239. ***Scapula***, angle between blade and acromion: gradual, oblique (0), abrupt, perpendicular (1).

240. ***Scapula***, size of acromion process: moderate (0), marked (1).

241. ***Scapula***, midshaft expansion of blade: absent (0), present (1).

242. ***Scapula***, distal expansion of blade: marked (0), weak/absent (1).

243. ***Scapula***, length:width ratio of blade: ≤ 7 (0), 7.5–9 (1), > 10 (2).

244. ***ScapuloCoracoid***, shape of anterior margin: indented or notched between acromial process and coracoid suture (0), smoothly curved and uninterrupted across scapula–coracoid contact (1).

245. ***ScapuloCoracoid***, glenoid lip: moderate (0), marked (1).

246. ***Coracoid***, development of posteroventral process: low, rounded posteroventral eminence (0), pronounced, posteroventrally tapering process (1).

247. ***Coracoid***, development of biceps tubercle (= acrocoracoid process): absent or poorly developed (0), conspicuous and well developed as tuber (1), developed as a posteroventrally oriented ridge (2).

248. ***Coracoid***, prominent fossa on ventral surface posteroventral to glenoid (subglenoid fossa): absent (0); present (1).

249. ***Humerus***, shape of head: elongate (0), globular (1).

250. ***Humerus***, longitudinal torsion of shaft: absent (0), present (1).

251. ***Humerus***, size of trochanters relative to midshaft diameter: < (0), > 150% (1) > 250% (2).

252. ***Humerus***, development of internal tuberosity: low/rounded (0), hypertrophied (1).

253. ***Humerus***, length of deltopectoral crest relative to total bone length: < 0.4 (0), 0.43–0.49 (1) > 0.52 (2).

254. ***Humerus***, height of deltopectoral crest: low (0), prominent, with gradually raised margin (1), prominent, with abruptly raised margin (2). (modified)

255. ***Humerus***, orientation of deltopectoral crest apex: anteriorly (0), anterolaterally (1).

256. ***Humerus***, relative orientation of proximal & distal condyles in anteroposterior view: parallel, humerus straight (0), distal canted (1).

257. ***Humerus***, anterior surface of bone adjacent to ulnar condyle: smooth or gently depressed (0), bears well-defined fossa (1).

258. ***Humerus***, shape of distal condyles: rounded (0), flattened (1).

259. ***Radius and Ulna***, development of radial external tuberosity and ulnar internal tuberosity: low, rounded (0), hypertrophied distal ends of radius and ulna broadened (1).

260. ***Radius***, shaft: straight (0); curves laterally (1).

261. ***Radius***, development of medial biceps tubercle: small or indistinct (0), hypertrophied (1).

262. olecranon process: absent (0), present (1).

263. ***Ulna***, morphology of olecranon process: transversely robust (0); transversely compressed and 'blade-like' (1).

264. ***Ulna***, crest extending distally along posterior surface from olecranon process: absent (0), present (1).

265. ***Ulna***, hypertrophied medial and lateral processes proximal end: absent (0), present (1).

266. ***Ulna***, length relative to minimum circumference: stout, < 2.3 (0); gracile > 2.6 (1).

267. ***Carpus***, morphology and articulations of distal carpals: separate dc1 and dc2 over separate metacarpals, flattened proximodistally (0), fused dc1 and dc2, dc1 overlaps metacarpals I and II, flattened proximodistally (1), fused dc1 and dc2, dc1 overlaps metacarpals I and II, strongly arched proximodistally (2).

268. ***Manus***, length relative to length of arm + forearm: < (0), ≥ (1).

269. ***Manus***, composition: digit IV and V present (0), digit IV present, digit V absent (1), MC IV present, IV phalanges and digit V absent (2), digits IV and V absent (3).

270. ***Manual digits***, lengths: III longest (0), II longest (1).

271. ***Metacarpals***, transverse width of proximal articular ends relative to minimum transverse shaft width: < (0), ≥ 2x (1).

272. ***Metacarpal I***, length to minimum width ratio: 1.4–1.9 (0), ≥ 2.4 (1).

273. ***Metacarpal I***, length relative to length of metacarpal II: > 50% (0), < 50% (1).

274. ***Metacarpal I***, extent of contact with metacarpal II relative to shaft length: < 1/3 (0), 1/2 (1).

275. ***Metacarpal I***, angle between facet for metacarpal II and proximal articular facet: perpendicular (0), obtuse (1).

276. ***Metacarpal III***, position of base relative to those of other metacarpals: at same level (0), on palmar surface (1).

277. ***Metacarpal III***, shape of proximal end: rectangular (0), triangular (1).

278. ***Metacarpal III***, width relative to width of metacarpal II: > 50% (0), < 50% (1).

279. ***Manual ungual I***, length:height ratio: < 2.5x (0), > 2.5x (1).

280. ***Manual unguals***, proximal height:width ratio: transversely broad, < 2.0 (0), transversely narrow, > 2.4 (1).

281. ***Pelvic elements***, articulations in adults: separate (0), fused (1).

282. ***Ilium***, large external pneumatic foramina and internal spaces: absent (0), present (1).

283. ***Ilium***, vertical ridge on lateral surface of blade dorsal to acetabulum: absent (0), low swollen ridge (1), low double ridge (2).

284. ***Ilium***, posterior width of brevis fossa: subequal to anterior width, fossa margins subparallel (0), twice anterior width, fossa widens posteriorly (1).

285. ***Ilium***, height of lateral wall of brevis fossa relative to medial wall: taller along whole length (0), shorter anteriorly, exposing medial wall in lateral view (1).

286. ***Ilium***, morphology between supraacetabular crest and brevis shelf on lateral surface: gap (0), continuous ridge (1).

287. ***Ilium***, ventrolateral development of supraacetabular crest: large/pendant 'hood' (0), reduced shelf (1).

288. ***Ilium***, orientation of pubic peduncle: mostly ventral (0), mostly anterior or 'kinked' double facet with anterior and ventral components (1).

289. ***Ilium***, shape of acetabular margin of pubic peduncle: transversely convex or flat (0); transversely concave (1)

290. ***Ilium***, relative sizes of pubic and ischial articulations: subequal (0), pubic articulation ≥ 130% of iliac articulation (1).

291. ***Ilium***, morphology of ischial peduncle: rounded (0), acuminate (1).

292. ***Ilium***, pubic peduncle length to width ratio: ≤ 1 (0), 1.3–1.75 (1), > 2 (2).

293. ***Ilium***, ridge on medial surface adjacent to preacetabular notch: absent (0), present (1), strongly developed, forming a shelf (2).

294. ***Ilium***, preacetabulum length relative to anterior edge of pubic peduncle: reaches anteriorly to same point as ('brachyiliac') (0), or well past ('dolichoiliac') (1).

295. ***Ilium***, depth of preacetabular process: shallow (0), deep (1).

296. ***Ilium***, anteroventral lobe of preacetabular process: absent (0), present (1).

297. ***Ilium***, shape of dorsal margin: convex (0), straight (1).

298. ***Ilium***, postacetabulum length relative to ischial peduncle length: ≤ (0), > (1), 2x (2).

299. ***Ilium***, depth of postacetabular process: shallow (0), deep (1).

300. ***Ilium***, shape of posterior margin of postacetabular process: convex (0), concave (1), straight (2), with prominent posterodorsal process but lacking posteroventral process (3).

301. ***Puboischiadic plate***: fully closed along midline (0), open along midline (1). (modified)

302. ***Pubis***, shaft orientation: straight (0), ventrally curved (1).

303. ***Pubis***, articulation between apices in adults: unfused (0); fused (1).

304. ***Pubis***, contact between distal portions: separate distally (0), contacting (1), contacting with slit-like opening proximal to distal expansion (interpubic fenestra) (2).

305. ***Pubis***, angle between long axes of shaft and boot: 75-90° (0), < 60° (1).

306. ***Pubis***, morphology of symphysis: marginal (0), broad (1).

307. ***Pubis***, morphology of obturator foramen: fully surrounded by bone (0), partly ventrally notched (1), totally missing ventral border. (modified)

308. ***Pubis***, anterior expansion of distal end: absent (0), present (1).

309. ***Pubis***, boot length: approximate to anteroposterior width of pubic shaft (0), slightly anteroposteriorly expanded (1), around 30% (1), around 60% (2). (modified)

310. ***Pubis***, shape of boot in ventral view: broadly triangular (0), narrow, with subparallel margins (1).

311. ***Pubis***, articulation with ilium: planoconcave (0), peg and-socket (1).

312. ***Ischium***, length relative to pubis length: 75–80% (0), ≤ 70% (1), > 80% (2).

313. ***Ischium***, shaft orientation: straight (0), ventrally

curved (1).

314. ***Ischium***, articulation with ilium: planoconcave (0), pegand-socket (1).

315. ***Ischium***, morphology of antitrochanter: large and notched (0), reduced (1).

316. ***Ischium***, notch ventral to obturator process: absent (0), present (1).

317. ***Ischium***, morphology of symphysis: unexpanded (0), expanded as apron (1).

318. ***Ischium***, cross-sectional shape of paired midshafts: oval (0), heart-shaped, medial portions of shafts extend posteriorly as midline flange (1).

319. ***Ischium***, morphology of distal end: does not expand, as anteroposteriorly wide as diameter of main shaft (0), slightly expanded (1), hook-like (2), tapering (3). (modified)

320. ***Ischium***, articulation at distal end in adults: separate (0), fused (1).

321. ***Ischium***, morphology of obturator foramen: absent or very small emargination (0), fully surrounded by bone (1), notched (2). (new)

322. ***Femur***, head orientation: 45° anteromedial (0), 10–30° anteromedial (1), medial (2).

323. ***Femur***, head angle: ventromedial (0), horizontal (medial) (1), dorsomedial (2).

324. ***Femur***, groove on proximal surface of head-oriented oblique to long axis of head (‘articular groove’): absent (0), present (1).

325. ***Femur***, oblique ligament groove on posterior surface of head: shallow, groove bounding lip does not extend past posterior surface of head (0), deep, bound medially by well-developed posterior lip (1).

326. ***Femur***, placement of lesser trochanter relative to femoral head: does not reach ventral margin (0), rises past ventral margin (1), rises to proximal surface (2).

327. ***Femur***, morphology of anterolateral muscle attachments at proximal end: continuous trochanteric shelf (0), distinct lesser trochanter and attachment bulge (1).

328. ***Femur***, development of fourth trochanter: prominent semioval flange (0), very weak or absent (1).

329. ***Femur***, distinctly projecting accessory trochanter (derived from lesser trochanter): weak, forms slightly thickened margin of lesser trochanter (0), present as triangular flange (1).

330. ***Femur***, M. femorotibialis externus origin medially on anterodistal surface: faint, small rugose patch (0), pronounced rugose depression that extends to distal femur (1).

331. ***Femur***, development of medial epicondyle: rounded (0), ridge (1).

332. ***Femur***, distal extensor groove: absent (0), present (1).

333. ***Femur***, morphology and orientation of tibiofibularis crest: broad (0), narrow, longitudinal (1), lobular, oblique (2).

334. ***Femur***, infrapopliteal ridge connecting medial distal condyle and crista tibiofibularis: absent (0), present (1).

335. ***Femur***, orientation of long axis of medial condyle in distal view: anteroposterior (0), posterolateral (1).

336. ***Femur***, projection of lateral and medial distal condyles: approximately equal (0), lateral projects distinctly further than medial, distal surface of medial is gently flattened (1).

337. ***Femur***, morphology of distal end: central depression connected to crista tibiofibularis by a narrow groove (0), anteroposteriorly oriented shallow trough separating medial and lateral convexities (1).

338. ***Tibia***, lateral malleolus: backs astragalus (0), overlaps calcaneum (1).

339. ***Tibia***, shape of edge of lateral malleolus: smoothly curved (0), tabular notch (1).

340. ***Tibia***, morphology of distal cnemial process: rounded (0), expanded proximodistally (1)

341. ***Tibia***, morphology of lateral (fibular) condyle: large (0), small and lobular (1).

342. ***Tibia***, anterolateral process of lateral condyle: absent or horizontal projection (0), prominent, curves

ventrally (1).

343. ***Tibia***, anteromedial buttress for astragalus: absent (0), ventral (1), marked oblique step-like ridge (2), reduced oblique ridge (3), bluntly rounded vertical ridge on medial side (4).

344. ***Tibia***, morphology of fibular crest: narrow (0), bulbous (1).

345. ***Tibia***, development of fibular crest: extends to proximal end of tibia as high crest (0), extends to proximal end of tibia as low ridge (1), does not extend to proximal end of tibia (2).

346. ***Fibula***, depth of fibular fossa on medial aspect: groove (0), shallow fossa (1), deep fossa (2).

347. ***Fibula***, position of fibular fossa on medial aspect: posterior edge (0), central (1).

348. ***Fibula***, size of iliofibularis tubercle: faint scar (0), large (1), anterolaterally curving flange (2).

349. ***Fibula***, size of proximal end relative to width of proximal tibia: < 75% (0), ≥ 75% (1).

350. ***Astragalus***, articulation between ascending process and fibula in adults: separate (0), fused (1).

351. ***Astragalus***, orientation of distal condyles: ventral (0), 30-45° anterior (1).

352. ***Astragalus***, ascending process morphology: blocky (0), laminar (1).

353. ***Astragalus***, angle of dorsal margin of ascending process: low and oblique (0), high and oblique (1).

354. ***Astragalus***, ascending process height relative to depth of astragular body: less (0), subequal (1), > 1.6 times (2).

355. ***Astragalus***, prominent proximolateral extension: absent (0); present (1).

356. ***Astragalus***, round fossa at base of ascending process: absent (0), small (1), large (2).

357. ***Astragalus***, development of articular surface for distal end of fibula: large, dorsal (0), reduced, lateral (1).

358. ***Astragalus***, posterolateral crest: absent (0), present (1).

359. ***Astragalus***, posteromedial crest: absent (0), present (1).

360. ***Astragalus***, articulation with calcaneum in adults: separate (0), fused (1).

361. ***Metatarsal I***, length relative to length of metatarsal II: ≥ 50% (0), < 50% (1).

362. ***Metatarsal III***, shape of proximal end: rectangular (0), shallow notch (1), deep notch (2).

363. ***Metatarsal III***, midshaft cross-sectional shape: rectangular (0), wedge-shaped, plantar surface pinched (1).

364. ***Metatarsal III***, relative proportions of shaft: short and thick, length: transverse width ratio < 12.0 (0), long and gracile, ratio > 12.5 (1).

365. ***Metatarsal IV***, proportions of distal end: broader than tall (0), taller than broad (1).

366. ***Metatarsal V***, morphology of distal end: articular (0), non-articular (1).

367. ***Metatarsal V***, length relative to length of metatarsal IV: > 50% (0), < 50% (1).

368. ***Antarctometatarsus***: absent (0), present (1).

369. ***Pedal unguals***, morphology of lateral and medial grooves: single (0), double (1).

370. ***Pedal unguals***, digits III and IV, cross-sectional shape: triangular (0), elliptical (1).

371. ***Pedal unguals***, digit II, mediolateral symmetry: symmetrical (0), asymmetrical (1).

372. ***Pedal digit phalanges***, length of I-1 + I-2 relative to III-1: greater (0), less than or equal (1).

Revision of characters from the Matrix of Carrano et al. (2012)

Character 9:

Eoraptor ? → 0

Acrocanthosaurus ? → 0

Duriavenator ? → 0

Monolophosaurus ? → 0

Character 19:

Sinraptor dongi 0 → 1

Character 24:

Herrerasaurus 0 → 1

Allosaurus 0 → 1

Baryonyx 0 → ?

Carcharodontosaurus 0 → 1

Compsognathus 1 → 0

Fukuiraptor 0 → ?

Giganotosaurus 0 → 1

Irritator 0 → 1

Majungasaurus 0 → 1

Mapusaurus 0 → 1

Marshosaurus 1 → 0

Monolophosaurus 0 → 1

Neovenator 0 → 1

Shaochilong 0 → 1

Sinraptor dongi 1 → 0

Sinraptor hepingensis 1 → 0

Spinosaurus 0 → 1

Suchomimus 0 → 1

'Szechuanosaurus.' zigongensis ? → 0

Torvosaurus 0 → 1

Character 25:

Irritator 1 → 0

Character 26:

Eoraptor 1 → 0

Coelophysis bauri 0 → 2

Coelophysis rhodesiensis 0 → 2

Fukuiraptor 0 → ?

Monolophosaurus 0 → 1

Proceratosaurus 0 → 2

Character 53:

Acrocanthosaurus 1 → 0

Monolophosaurus 1 → 0

Character 54:

Herrerasaurus 2 → 0

Carcharodontosaurus ? → 2

Concavenator 0 → 2

Monolophosaurus 2 → 0

Ornitholestes 2 → 0

Proceratosaurus 2 → 0

Character 64:

Carcharodontosaurus ? → 2

Concavenator 1 → 2

Saurophaganax 0 → ?

Character 65:

Herrerasaurus 0 → 1

Proceratosaurus 0 → 1

Character 67:

Eocarcharia 0 → 1

Character 75:

Ornitholestes ? → 0

Character 90:

Ornitholestes 0 → 1

Character 98:

Acrocanthosaurus ? → 1

Giganotosaurus ? → 1

Mapusaurus ? → 1

Character 99:

Eoraptor 0 → ?

Character 126:

Acrocanthosaurus 1 → 0

Character 136:

Neovenator 1 → 0

Australovenator ? → 0

Character 137:

Australovenator ? → 0

Character 142:

Australovenator 1 → ?

Character 171:

Irritator ? → 2

Spinosaurus ? → 1

Suchomimus 1 → 0

Character 180:

Herrerasaurus ? → 1

Baryonyx 1 → 0

Ceratosaurus 0 → 1

Dilophosaurus ? → 0

Majungasaurus 0 → 1

Mapusaurus ? → 0

Masiakasaurus 0 → 1

Character 183:

Concavenator ? → 1

Leshansaurus 1 → 0

Neovenator ? → 1

Character 203:

Shidaisaurus 1 → 0

Character 207:

Aerosteon 1 → 0

Character 219:

Concavenator 1 → 0

Character 244:

Masiakasaurus 1 → 0

Megalosaurus ? → 0

Majungasaurus ? → 1

Character 249:

Australovenator ? → 0

Character 250:

Australovenator ? → 1

Concavenator ? → 1

Character 251:

Australovenator ? → 2

Character 252:

Australovenator ? → 1

Chilantaisaurus 0 → 1

Character 253:

Australovenator ? → 0

Chilantaisaurus 1 → 0

Ornitholestes 1 → 0

Character 255:

Australovenator ? → 0

Character 256:

Australovenator ? → 1

Character 257:

Australovenator ? → 1

Character 258:

Australovenator ? → 0

Character 261:

Megalosaurus 0 → 1

Character 268:

Majungasaurus ? → 0

Character 269:

Majungasaurus ? → 1

Ornitholestes 1 → 2

Character 287:

Shidaisaurus 1 → 0

Sinraptor dongi 1 → 0

Sinraptor hepingensis 1 → 0

Yangchuanosaurus 1 → 0

CNM V214 1 → 0

Character 302:

Ceratosaurus 0 → 1

Coelophysis rhodesiensis 0 → 1

Character 308:

Ceratosaurus 0 → 1

Leshansaurus ? → 0

Siamotyrannus 0 → 1

Character 312:

Eoraptor 0 → 1

Marshosaurus ? → 0

Character 313:

Ornitholestes 0 → 1

Character 316:

Coelophysis rhodesiensis 0 → 1

Concavenator ? → 1

Yangchuanosaurus 1 → 0

Character 317:

Herrerasaurus ? → 0

Character 326:

Yangchuanosaurus 0 → 1

Character 336:

Chilantaisaurus ? → 1

Leshansaurus 1 → 0

Character 346:

Elaphrosaurus ? → 2

References

Allain R. 2002. Discovery of megalosaur (Dinosauria, Theropoda) in the middle Bathonian of Normandy (France) and its implications for the phylogeny of basal Tetanurae. *Journal of Vertebrate Paleontology* **22(3)**:548-563. https://doi.org/10.1671/0272-4634(2002)022[0548:Domdti]2.0.Co;2

Benson RBJ. 2010. A description of *Megalosaurus bucklandii* (Dinosauria: Theropoda) from the Bathonian of the UK and the relationships of Middle Jurassic theropods. *Zoological Journal of the Linnean Society* **158(4)**:882-935. https://doi.org/10.1111/j.1096-3642.2009.00569.x

Benson RBJ, Carrano MT, Brusatte SL. 2010. A new clade of archaic large-bodied predatory dinosaurs (Theropoda: Allosauroidea) that survived to the latest Mesozoic. *Naturwissenschaften* **97(1)**:71-78. https://doi.org/10.1007/s00114-009-0614-x

Brusatte SL, Norell MA, Carr TD, Erickson GM, Hutchinson JR, Balanoff AM, Bever GS, Choiniere JN, Makovicky PJ, Xu X. 2010. Tyrannosaur Paleobiology: New Research on Ancient Exemplar Organisms. *Science* **329(5998)**:1481-1485. https://doi.org/10.1126/science.1193304

Brusatte SL, Sereno PC. 2008. Phylogeny of Allosauroidea (Dinosauria: Theropoda): Comparative analysis and resolution. *Journal of Systematic Palaeontology* **6(2)**:155-182. https://doi.org/10.1017/s1477201907002404

Carrano MT, Benson RBJ, Sampson SD. 2012. The phylogeny of Tetanurae (Dinosauria: Theropoda). *Journal of Systematic Palaeontology* **10(2)**:211-300. https://doi.org/10.1080/14772019.2011.630927

Coria RA, Currie PJ. 2002. The braincase of *Giganotosaurus carolinii* (Dinosauria: Theropoda) from the Upper Cretaceous of Argentina. *Journal of Vertebrate Paleontology* **22(4)**:802-811. https://doi.org/10.1671/0272-4634(2002)022[0802:Tbogcd]2.0.Co;2

Eddy DR, Clarke JA. 2011. New information on the cranial anatomy of *Acrocanthosaurus atokensis* and its implications for the phylogeny of Allosauroidea (Dinosauria: Theropoda). *PLoS One* **6(3)**:e17932. https://doi.org/10.1371/journal.pone.0017932

Forster CA. 1999. Gondwanan dinosaur evolution and biogeographic analysis. *Journal of African Earth Sciences* **28(1)**:169-185. https://doi.org/10.1016/S0899-5362(99)00023-8

Gauthier J. 1986. Saurischian monophyly and the origin of birds. *Memoirs of the California Academy of Science* **8**:1-55.

Harris JD. 1998. *A reanalysis of Acrocanthosaurus atokensis, its phylogenetic status, and paleobiogeographic implications, based on a new specimen from Texas*: New Mexico Museum of Natural History.

Holtz TR. 1998. A new phylogeny of the carnivorous dinosaurs. *Gaia* **15**:5-61.

Holtz TRJ, Molnar RE, Currie PJ. 2004. Basal Tetanurae. In: David BW, Peter D, Halszka O, eds. *The dinosauria 2nd edition*, 71-110.

Lamanna MC, Casal GA, Martínez RDF, Ibiricu LM. 2020. Megaraptorid (Theropoda: Tetanurae) Partial Skeletons from the Upper Cretaceous Bajo Barreal Formation of Central Patagonia, Argentina: Implications for the Evolution of Large Body Size in Gondwanan Megaraptorans. *Annals of Carnegie Museum* **86(3)**:255-294. https://doi.org/10.2992/007.086.0302

Novas FE, Agnolín FL, Ezcurra MD, Porfiri J, Canale JI. 2013. Evolution of the carnivorous dinosaurs during the Cretaceous: The evidence from Patagonia. *Cretaceous Research* **45**:174-215. https://doi.org/10.1016/j.cretres.2013.04.001

Rauhut OWM. 2003. *The interrelationships and evolution of basal theropod dinosaurs*. London: The palaeontological association.

Schade M, Rauhut OWM, Foth C, Moleman O, Evers SW. 2023. A reappraisal of the cranial and mandibular

osteology of the spinosaurid *Irritator challengeri* (Dinosauria: Theropoda). *Palaeontologia Electronica* **26(2)**:1-116. https://doi.org/10.26879/1242

Smith ND, Makovicky PJ, Hammer WR, Currie PJ. 2007. Osteology of *Cryolophosaurus ellioti* (Dinosauria: Theropoda) from the Early Jurassic of Antarctica and implications for early theropod evolution. *Zoological Journal of the Linnean Society* **151(2)**:377-421. https://doi.org/10.1111/j.1096-3642.2007.00325.x
