# Supplementary material for: A new metriacanthosaurid theropod dinosaur from the Middle Jurassic of Yunnan Province, China
Source: PeerJ. 2025 Apr 2;13:e19218. doi: 10.7717/peerj.19218 (PMC11971988; doi:10.7717/peerj.19218)

Supplemental File S2

Characters mapping of the equally weighting strict consensus tree, all character numbers should be added one, becaurse this is exported directly from TNT v1.6.

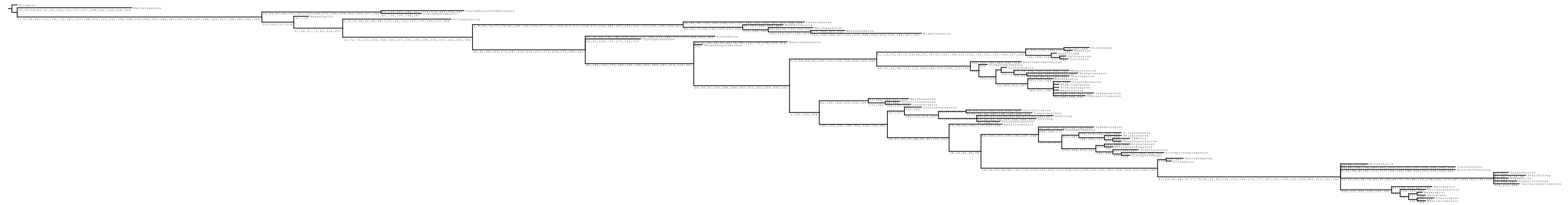

Supplement: Supplemental Information 2 [file peerj-13-19218-s002.pdf]
