# Supplementary figures and images for: A new metriacanthosaurid theropod dinosaur from the Middle Jurassic of Yunnan Province, China
Source: PeerJ. 2025 Apr 2;13:e19218. doi: 10.7717/peerj.19218 (PMC11971988; doi:10.7717/peerj.19218)

# Supplemental File S3

## Results of implied weighting analyses under different values of k

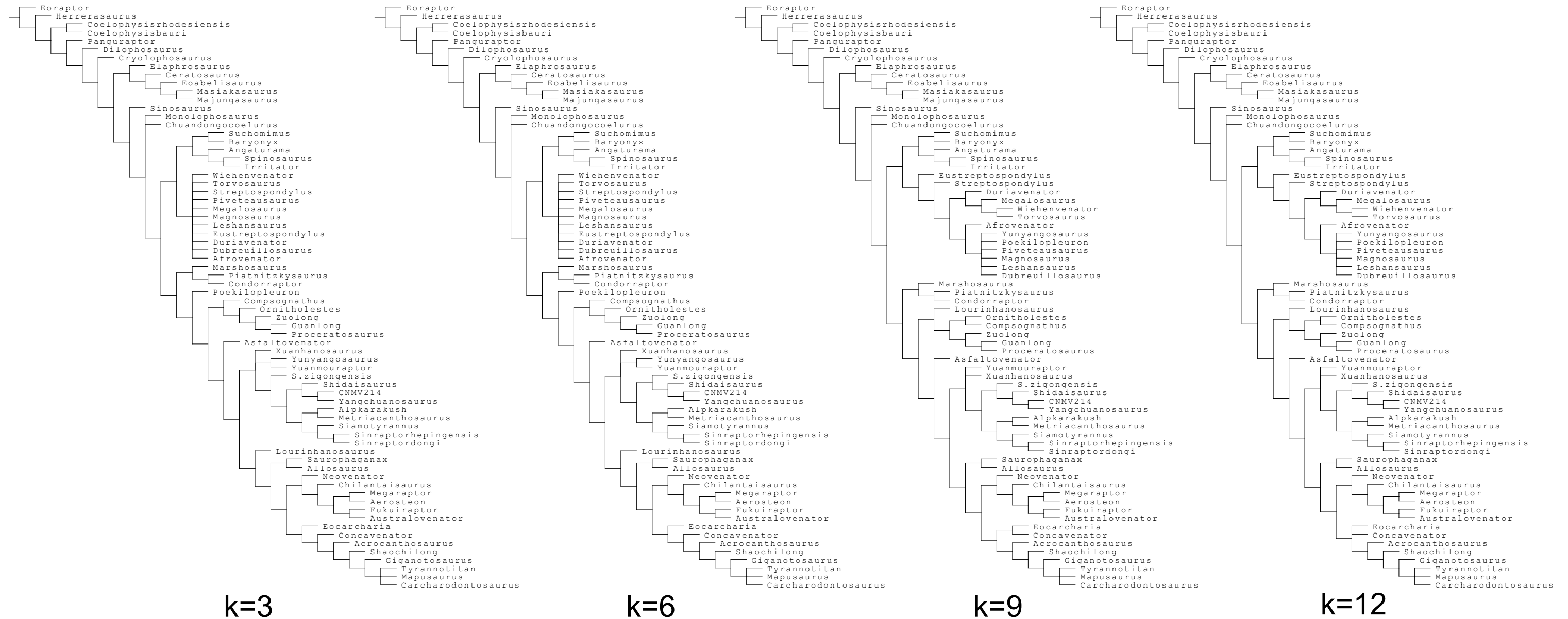

Supplement: Supplemental Information 3 [file peerj-13-19218-s003.pdf]

# Supplemental File S5

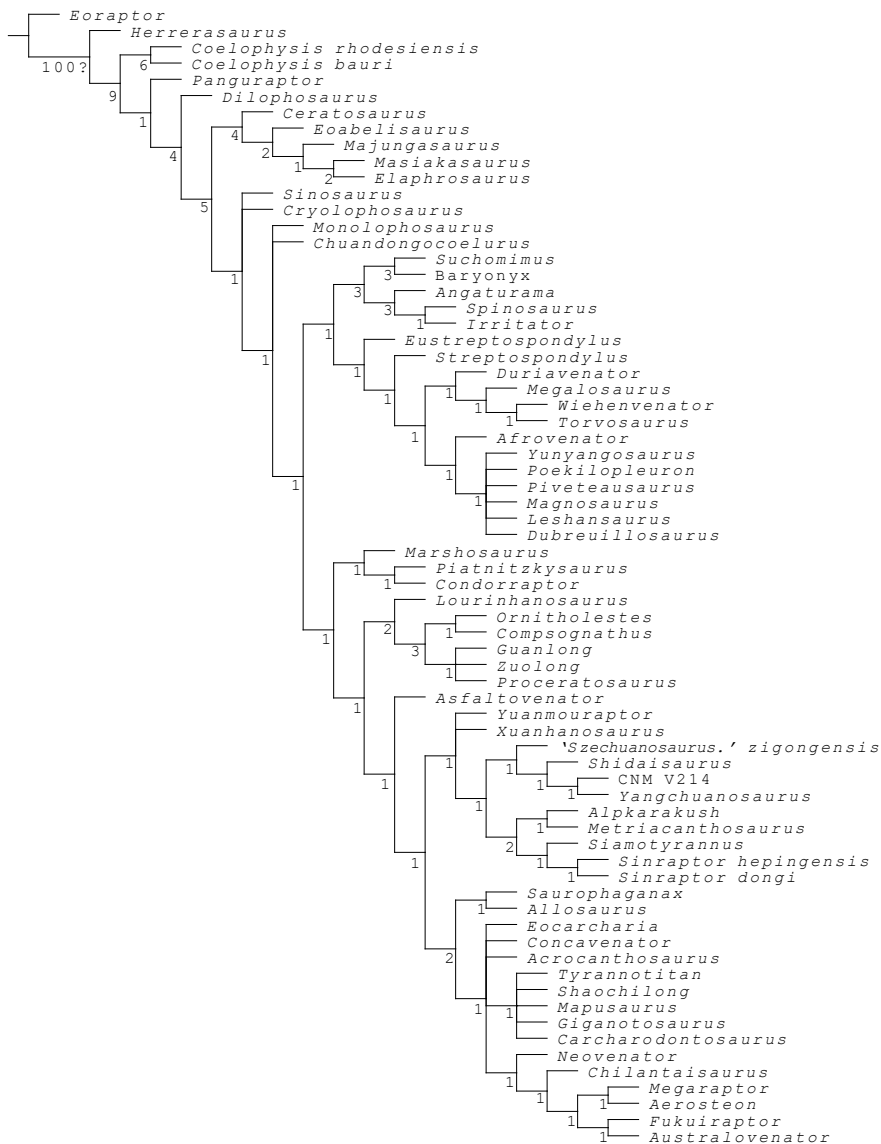

Supplement: Supplemental Information 5 [file peerj-13-19218-s005.pdf]

# Supplemental File S6

Group freqs., 10000 replicates, cut=50 (tree 0) - Standard Bootstrap

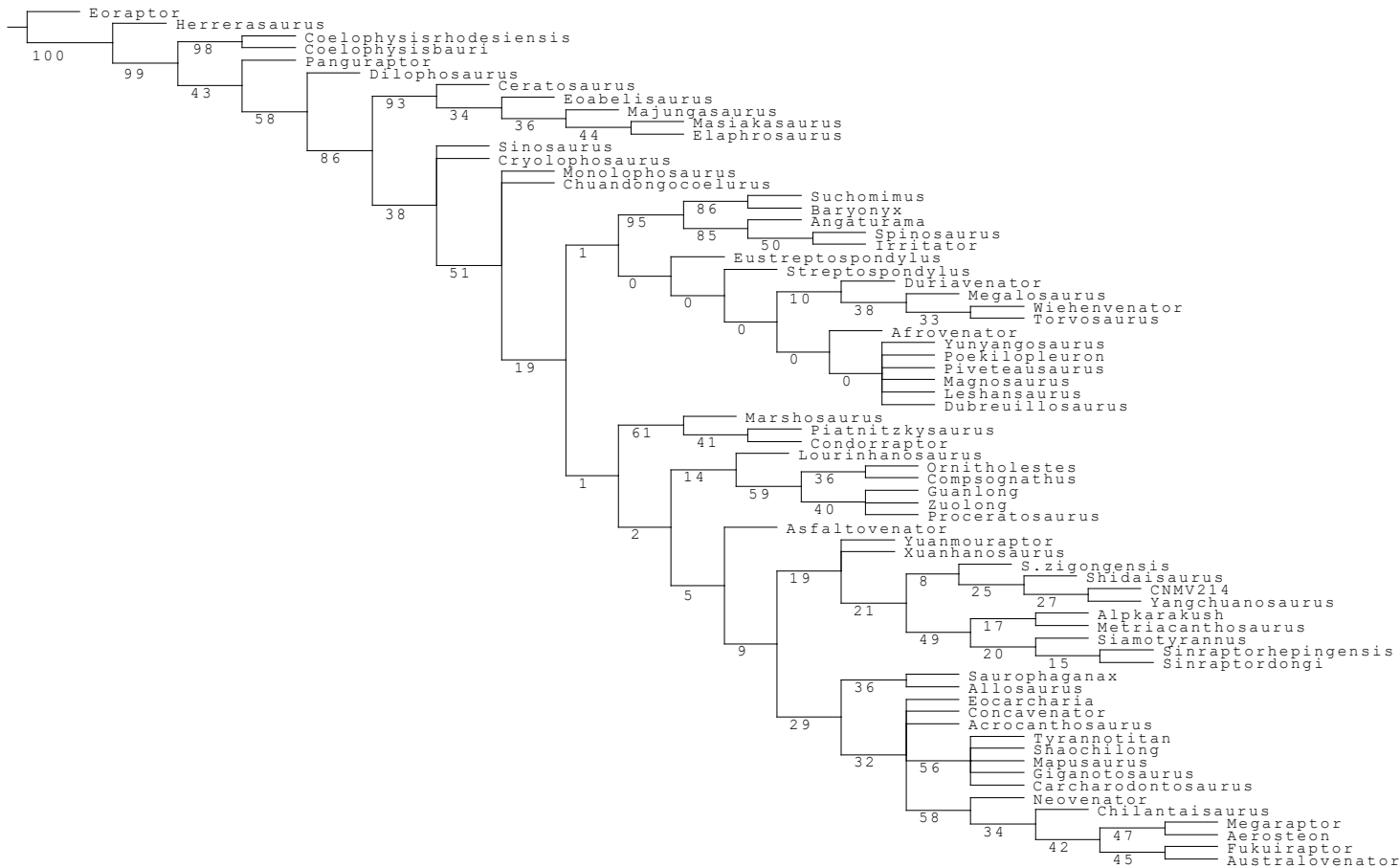

Supplement: Supplemental Information 6 [file peerj-13-19218-s006.pdf]
