# Supplementary material for: A new metriacanthosaurid theropod dinosaur from the Middle Jurassic of Yunnan Province, China
Source: PeerJ. 2025 Apr 2;13:e19218. doi: 10.7717/peerj.19218 (PMC11971988; doi:10.7717/peerj.19218)

Supplemental File S4

Unconstraint analysis

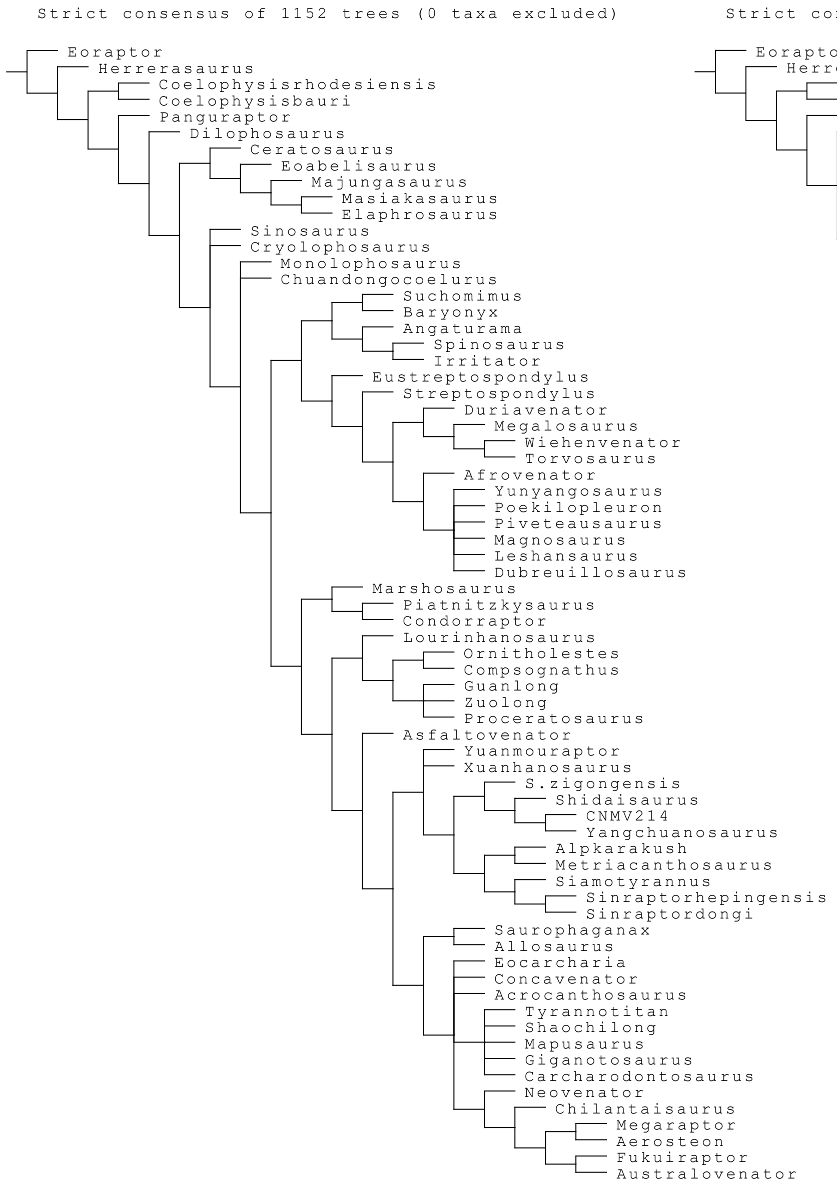

Topology 1

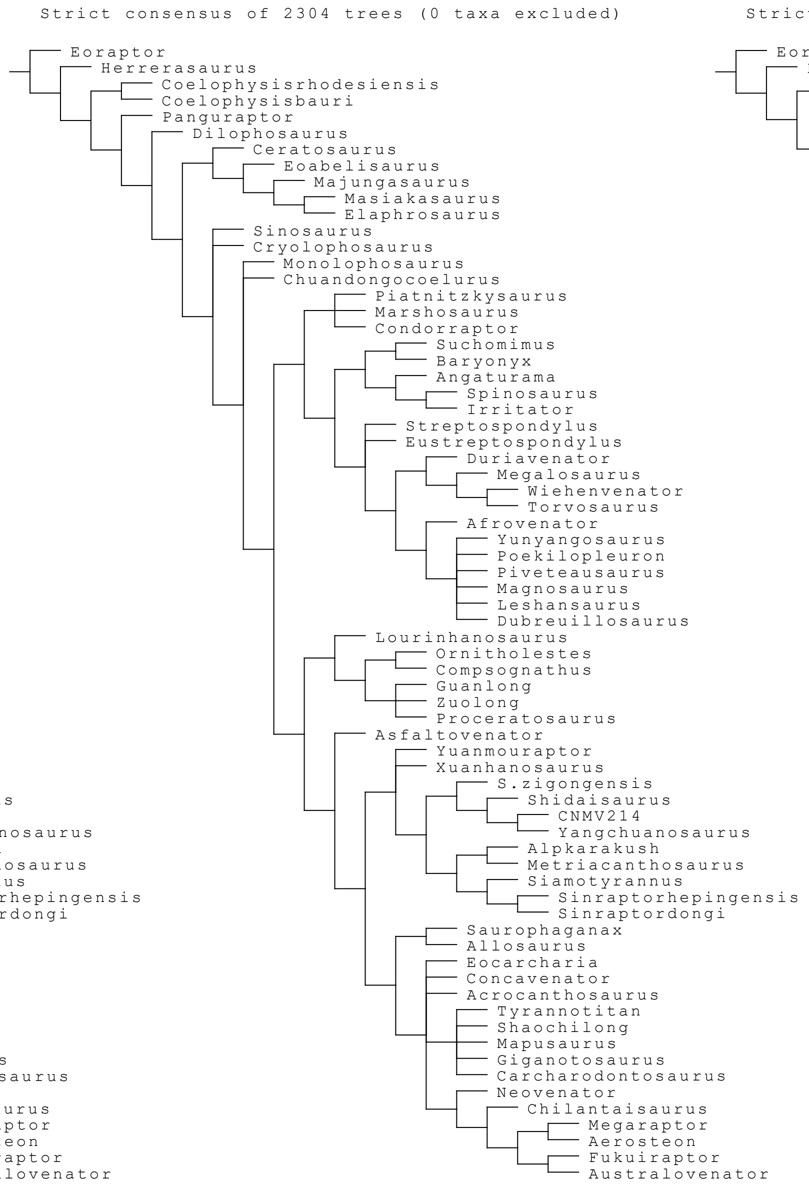

Topology 2

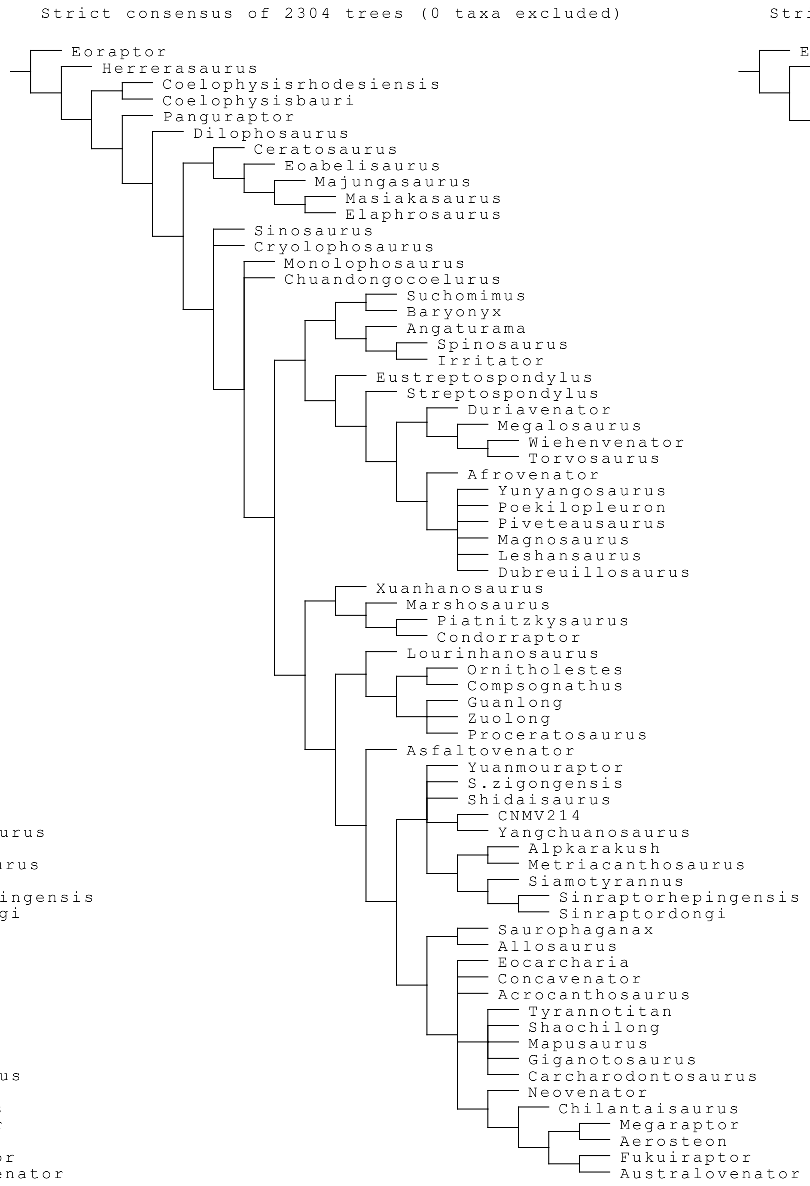

Topology 3

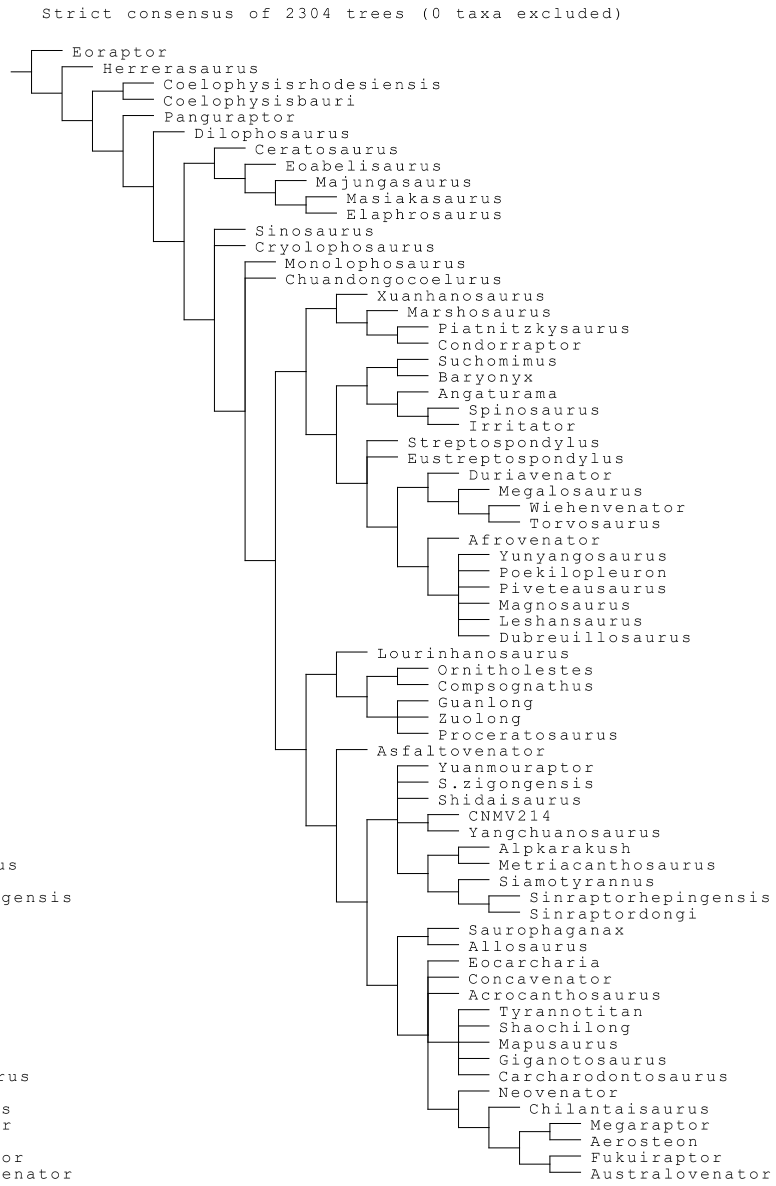

Supplement: Supplemental Information 4 [file peerj-13-19218-s004.pdf]
